# Two-color bursting oscillations

**DOI:** 10.1038/s41598-017-08751-y

**Published:** 2017-08-21

**Authors:** Bryan Kelleher, Bogusław Tykalewicz, David Goulding, Nikita Fedorov, Ilya Dubinkin, Thomas Erneux, Evgeny A. Viktorov

**Affiliations:** 1 Department of Physics, University College Cork, Cork, Ireland; 2 Tyndall National Institute, University College Cork, Lee Maltings, Dyke Parade, Cork, Ireland; 3 Centre for Advanced Photonics and Process Analysis, Cork Institute of Technology, Cork, Ireland; 4 National Research University of Information Technologies, Mechanics and Optics, Saint Petersburg, Russia; 5 Optique Nonlinéaire Théorique, Campus Plaine, CP 231, 1050 Bruxelles, Belgium

## Abstract

Neurons communicate by brief bursts of spikes separated by silent phases and information may be encoded into the burst duration or through the structure of the interspike intervals. Inspired by the importance of bursting activities in neuronal computation, we have investigated the bursting oscillations of an optically injected quantum dot laser. We find experimentally that the laser periodically switches between two distinct operating states with distinct optical frequencies exhibiting either fast oscillatory or nearly steady state evolutions (two-color bursting oscillations). The conditions for their emergence and their control are analyzed by systematic simulations of the laser rate equations. By projecting the bursting solution onto the bifurcation diagram of a fast subsystem, we show how a specific hysteresis phenomenon explains the transitions between active and silent phases. Since size-controlled bursts can contain more information content than single spikes our results open the way to new forms of neuron inspired optical communication.

## Introduction

Neurons communicate by repetitive firing or by brief bursts of spikes separated by silent periods. Two biophysical mechanisms are needed to produce these bursting activities. One mechanism generates either a fast pulsating state or a nearly steady state while the other mechanism produces a slow modulation that causes switches between the silent and active phases. Bursting oscillations are not limited to neurons and appear in a large variety of excitable cells such as the *β*–cells secreting insulin in response to glucose. While different forms of biological bursting outputs have been studied mathematically since the end of the eighties^1–3^, bursting phenomena are less documented in the laser literature. In refs 4–6, multiple intensity spikes separated intermittently by quasi-steady time intervals were observed for CO_2_ lasers with different saturable absorbers and later explained by identifying a slow variable that modulates a fast subsystem of equations^7^ (p 199). Bursting oscillations resulting from a slow/fast process were also observed and analyzed for an optoelectronic oscillator subject to a long delayed feedback^7^ (p 261), ref. 8. Thermal processes are usually ignored in laser optics but, because of their slow timescale, they may act as the slow modulator in bursting phenomena. This is the case for degenerate optical parametric oscillators where frequency detunings are slowly varying functions of the temperature^7, 9^ (p 307). In this work, we show that a slow thermal process plays a similar role in an injected dual-state quantum dot (QD) laser. Slow changes in temperature lead to slow modulations of the frequency detuning between master and slave lasers which then periodically initiate intervals of active spikes. Properly controlled, these bursting oscillations can be considered as new forms of fast spike-time coding with potentially more informational content than single spikes when analyzed as unitary events. Information may be encoded into the burst duration or in the fine temporal structure of interspike intervals within a burst. In the context of information processing, neuroinspired networks of lasers delivering single spikes under supra threshold stimuli^10–14^ have already been tested and several systems exhibiting bursting oscillations have recently been proposed as possible candidates for neuromorphic systems^15–17^. Here, we propose a two time-scale analysis of the laser equations and identify the physical mechanism leading to the bursting oscillations. Specifically, we determine the bifurcation diagram of the fast subsystem and show how a particular hysteresis phenomenon explains the transitions between active and silent phases.

Uniquely in semiconductor lasers, free running QD devices can lase from multiple distinct energy states. In particular, depending on the operating and device parameters, QD lasers can lase from the ground state (GS), from the first excited state (ES), and even simultaneously from both states. When the solitary laser operates in the ES and is then optically injected at the GS, large hysteresis cycles between pure ES and mixed ES + GS states are possible leading to bistable responses between steady and pulsating regimes^18, 19^. Provided the injection power is sufficiently high, switchings between high and low amplitude intensities promote slow increases or decreases of the detuning leading to dynamical hysteresis cycles. As we shall demonstrate experimentally and numerically, the switching between silent and fast oscillating phases leads to a new form of square-wave bursting oscillations, not yet documented in the literature^1–3^. Since the mid-eighties, considerable efforts have been made to classify the different types of bursters^20–22^. The list is necessarily incomplete due to the large variety of bifurcations in neural systems^1^. In our work, the emergence of bursting oscillations in the QD laser relies on its unique property to operate in distinct states. Specifically, we electrically pump the device so that it is emitting from the ES only when free-running and then we inject light into the GS and observe bursting oscillations in both the GS and ES exhibiting distinct optical frequencies (two color bursting). Importantly, we may experimentally control the size of the active phase by changing the injection strength.

## Results

In the experiment, the master laser (ML) was a commercially available tunable laser with a linewidth less than 100 kHz. It was tunable in steps of 0.1 pm. The slave laser was a 0.6 mm long InAs based QD laser similar to the one used in ref. 23. When emitting from the GS, the output was from a single longitudinal mode at approximately 1300 nm. When emitting from the ES, the output was multimode with a spectral width of approximately 10 nm centered close to 1220 nm. The threshold to lasing from the GS only was 34 mA. Between 60 mA and 80 mA, lasing occurred from both the GS and ES simultaneously. Above 80 mA emission was from the ES only. For all our experiments here the slave was biased at 84 mA. As in ref. 19 light from the ML was coupled to the SL via an optical circulator and lensed fibre with a polarization controller to ensure maximal coupling. The circulator Two tunable filters were used to allow simultaneous capture of the GS and ES outputs. Both signals were analyzed on a fast real time digital oscilloscope via fast photodetectors with an overall detection bandwidth of 15 GHz.

**Figure 1:**
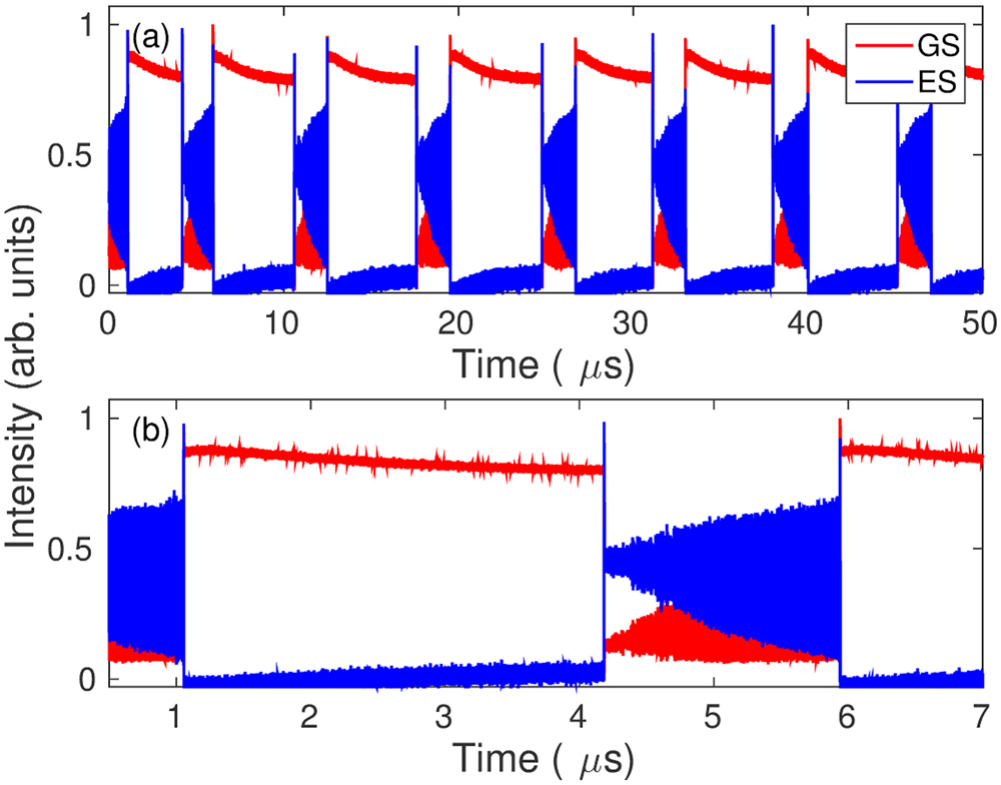
(**a**,**b**) Show experimental times traces. The upper panel (a) shows an example of the dropout and recovery phenomenon. The lower panel (b) shows a zoom of (**a**).

**Figure 2:**
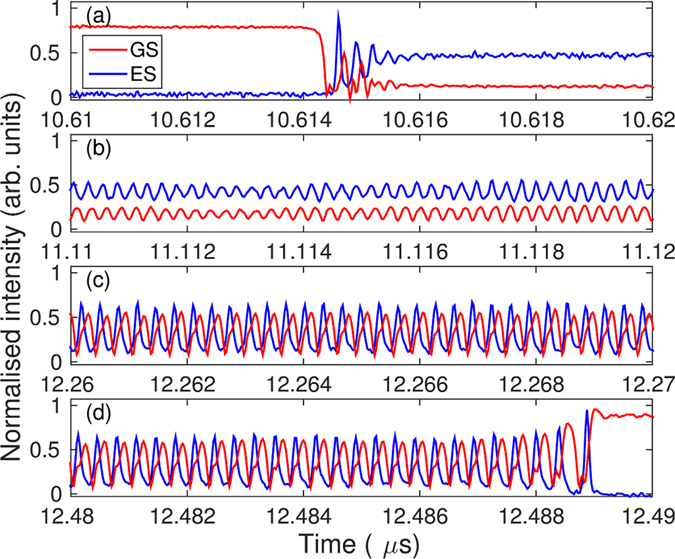
Experimental times traces showing four zooms of one of the dropouts of Fig. 1. The time axes show the positions of the zooms. (**a**) Shows the initial dropout and relaxation around the focus. (**b**–**d**) Show the evolution of the oscillations both in amplitude and shape until the eventual return to the high GS intensity and low ES intensity state.

In our previous work^18^, the wavelength of the ML was fixed and we observed hysteresis between the GS and ES states when the injection power was varied around a central value of approximately 0.5 mW. Similarly, the system exhibited hysteresis when the detuning was varied rather than the injection strength^19^. In this work, we operated the ML at a higher power of 2 mW. In this case, the hysteresis loop was not observed but rather we found bursts of oscillations separated by periods of quiescence. Figures 1 and 2 show the behavior of both the GS and ES intensities. The GS undergoes rapid dropouts followed by complex oscillations before switching back to a quasi-steady high intensity regime. Detailed zooms of the oscillating regime are shown in Fig. 2. The dropout is characterized by a few damped oscillations followed by a prolonged almost flat region (see Fig. 2(a)). After a critical time, growing oscillations appear and persist for approximately 1.5 *μ*s. The GS then jumps to an almost constant high intensity (see Fig. 2(b–d)). The evolution of the ES intensity is similar. It starts at approximately zero and then quickly grows (corresponding to the GS dropout) to a moderate level with damped oscillations. The ES intensity then follows the same evolution as the GS intensity although with its oscillations in antiphase to those of the GS. Finally, the ES intensity quickly drops back to approximately zero corresponding to the high intensity GS plateau. These observations suggest that we have uncovered a bursting cycle alternating between a large intensity GS state (the silent phase) and a lower intensity GS state where sustained oscillations are possible (the active phase). Numerical simulations of the laser rate equations described below substantiate the bursting cycle scenario. The simulations combined with a bifurcation analysis of a fast subsystem indicate that the oscillations appear via a slow passage through a Hopf bifurcation. Moreover, the down and up jumps between silent and active phases result from slow passages through a steady limit point and a limit point of limit-cycles, respectively.

**Figure 3:**
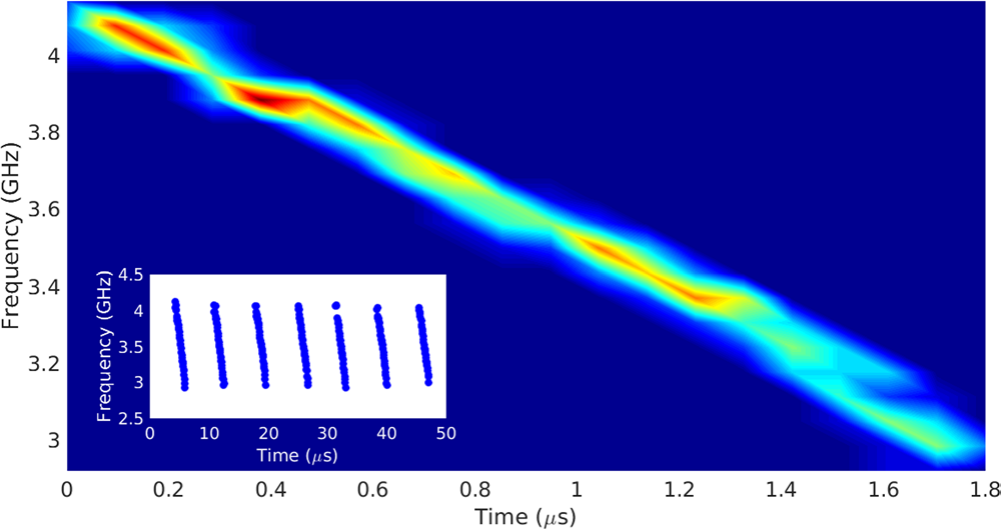
Experimental false colour histogram of the frequency evolution. The inset shows the individual frequency evolutions from consecutive bursts. By temporally shifting each of these so that they lie on top of each other one can build a two-dimensional histogram by creating bins in both the frequency and the temporal position. The resulting histogram is narrow and has a well-defined centre in each bin confirming the deterministic nature of the bursting trajectory.

We experimentally investigate the slow transition through the Hopf bifurcation by performing two tests. We first examine the behavior of the phase of the electric field along the low GS intensity regime. Using the phase resolving technique developed in ref. 24, we find that the phase remains bounded when the oscillations are growing, as expected from a Hopf bifurcation^25^. The second test concentrates on the frequency of the oscillations. In the conventional single mode injection system, the approximation of the Hopf bifurcation frequency, given by Eq. (9.54) in ref. 7, is ${\omega }_{H}=\sqrt{{\omega }_{R}^{2}+{\varepsilon }_{H}^{2}}$ where *ω*_*R*_ denotes the relaxation oscillation frequency of the solitary laser and *ε*_*H*_ is the injection rate at the Hopf bifurcation point. The latter is related to the detuning by Eq. (9.52) in^7^. For ε_H_ sufficiently large, ε_H_^2^ ~ Δ^2^. Therefore, a decrease of the magnitude of the detuning implies a decrease of *ω*_*H*_. Thus, if the magnitude of the detuning is decreasing, we should then observe a decrease of the oscillation frequency. To test this, we break the time evolution of the GS intensity oscillations into short sections and analyze the frequency contents by applying Fast Fourier Transforms. The results are shown in Fig. 3 where we perform a statistical analysis of the frequency evolution. We first measure the frequency evolution across each burst and plot the position of the main peak in the FFT for each section. This is shown in the inset and clearly demonstrates the decreasing frequency, agreeing with our interpretation. We then shift these individual lines temporally so that they all lie on top of each other. Finally, we then arrive at Fig. 3 by plotting a false-colour two-dimensional histogram with bins in the frequency and in the temporal position. The histogram is quite narrow and a central point is well-defined for each bin. The resulting figure acts similarly to a persistence trace on an oscilloscope showing the essentially unchanging evolution through the bursts. Thus we can conclude that each burst follows a deterministic trajectory once the oscillations begin. In addition, we have experimentally verified that the duration of the active phase can be controlled by changing the injection rate. We obtain durations from approximately 1 *μ*s to approximately 10 *μ*s.

In order to describe the different stages during a complete bursting cycle, we consider the rate equations formulated in ref. 19. The model takes into account the differing spin degeneracies in the QD energy levels, Pauli blocking, and interstate captures and escapes. Specifically, it consists of nine equations for the complex electric field of the GS (*E*_*g*_), the intensity of the ES (*I*_*e*_), the electron (hole) occupation probabilities of the GS $({n}_{e(h)}^{g})$ and ES $({n}_{e(h)}^{e})$ and the electron (hole) carrier density in the wetting layer $({n}_{e,h}^{w})$. In these equations, *α* is the phase-amplitude coupling (linewidth enhancement factor) which we take to be constant for simplicity. Similarly, the gain coefficients are considered equal and are represented by *g*. *β* models the effect of inhomogeneous broadening^19^, *ε* is the injection strength, and Δ = *ω*_*i*_ − *ω*_*g*_ is the detuning between the frequency of the injected light and that of the GS. *J* is the pump current. The capture rates to the GS and ES are given by *B*_*e*,*h*_ and ${B}_{e,h}^{w}$ respectively. The *C* terms determine the escape rates and are linked to the capture rates via a Kramers relation^18^. Our primary control parameter is the injection strength *ε*.

The equations are

1 $${E}_{g}^{^{\prime} }=\frac{1}{2}\,[\mathrm{(1}+i\alpha )\,\mathrm{(2}g({n}_{e}^{g}+{n}_{h}^{g}-\mathrm{1)}-\mathrm{1)}+4i\beta g({n}_{e}^{e}+{n}_{h}^{e}-\mathrm{1)}]\,{E}_{g}-i{\rm{\Delta }}{E}_{g}+\varepsilon ,$$

2 $${I}_{e}^{^{\prime} }=[4g({n}_{e}^{e}+{n}_{h}^{e}-\mathrm{1)}-1]\,{I}_{e},$$

3 $${n}_{e,h}^{g^{\prime} }=\eta [2{B}_{e,h}{n}_{e,h}^{e}\mathrm{(1}-{n}_{e,h}^{g})-2{C}_{e,h}{n}_{e,h}^{g}\mathrm{(1}-{n}_{e,h}^{e})-{n}_{e}^{g}{n}_{h}^{g}-g({n}_{e}^{g}+{n}_{h}^{g}-\mathrm{1)}\,{|{E}_{g}|}^{2}],$$

4 $$\begin{array}{rcl}{n}_{e,h}^{e^{\prime} } & = & \eta \,[-{B}_{e,h}{n}_{e,h}^{e}\mathrm{(1}-{n}_{e,h}^{g})+{C}_{e,h}{n}_{e,h}^{g}\mathrm{(1}-{n}_{e,h}^{e})+{B}_{e,h}^{w}{n}_{e,h}^{w}\mathrm{(1}-{n}_{e,h}^{e})\\  &  & -{C}_{e,h}^{w}{n}_{e,h}^{e}-{n}_{e}^{e}{n}_{h}^{e}-g({n}_{e}^{e}+{n}_{h}^{e}-\mathrm{1)}{I}_{e}],\end{array}$$

5 $${n}_{e,h}^{w^{\prime} }=\eta \,[J-{n}_{e}^{w}{n}_{h}^{w}-4{B}_{e,h}^{w}{n}_{e,h}^{w}\mathrm{(1}-{n}_{e,h}^{e})+4{C}_{e,h}^{w}{n}_{e,h}^{e}].$$

We next assume that thermal variations of the refractive index can be modeled as linear functions of the temperature and that they lead to variations of the phase of the GS electric field only via the detuning Δ. The rate of change of Δ is assumed to depend on the total intensity *I*_*g*_ + *I*_*e*_ where *I*_*g*_ = |*E*_*g*_|^2^ and *I*_*e*_ are the intensities of the GS and the ES, respectively. As for the case of the optical parametric oscillator^9^, the evolution of the detuning is then described by a first order differential equation of the form

6 $${\gamma }_{th}^{-1}{\rm{\Delta }}^{\prime} ={{\rm{\Delta }}}_{0}-{\rm{\Delta }}+c({I}_{g}+{I}_{e}).$$

**Figure 4:**
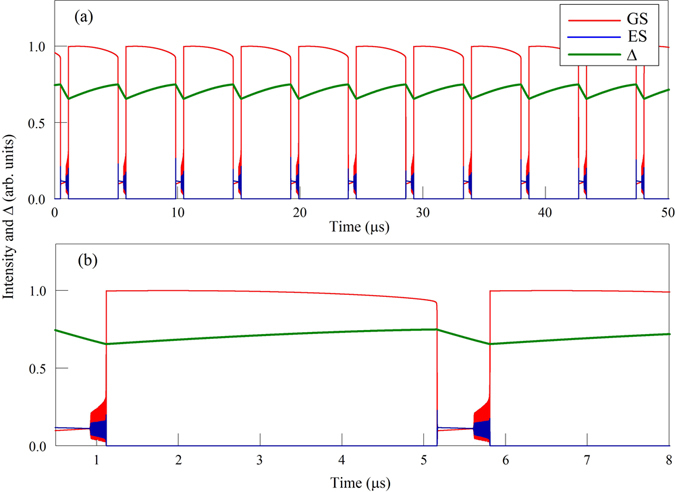
Numerical times traces. The upper panel (a) shows an example of the dropout and recovery phenomenon with the evolution of the detuning overlaid. The lower panel (b) shows a zoom of (**a**).

**Figure 5:**
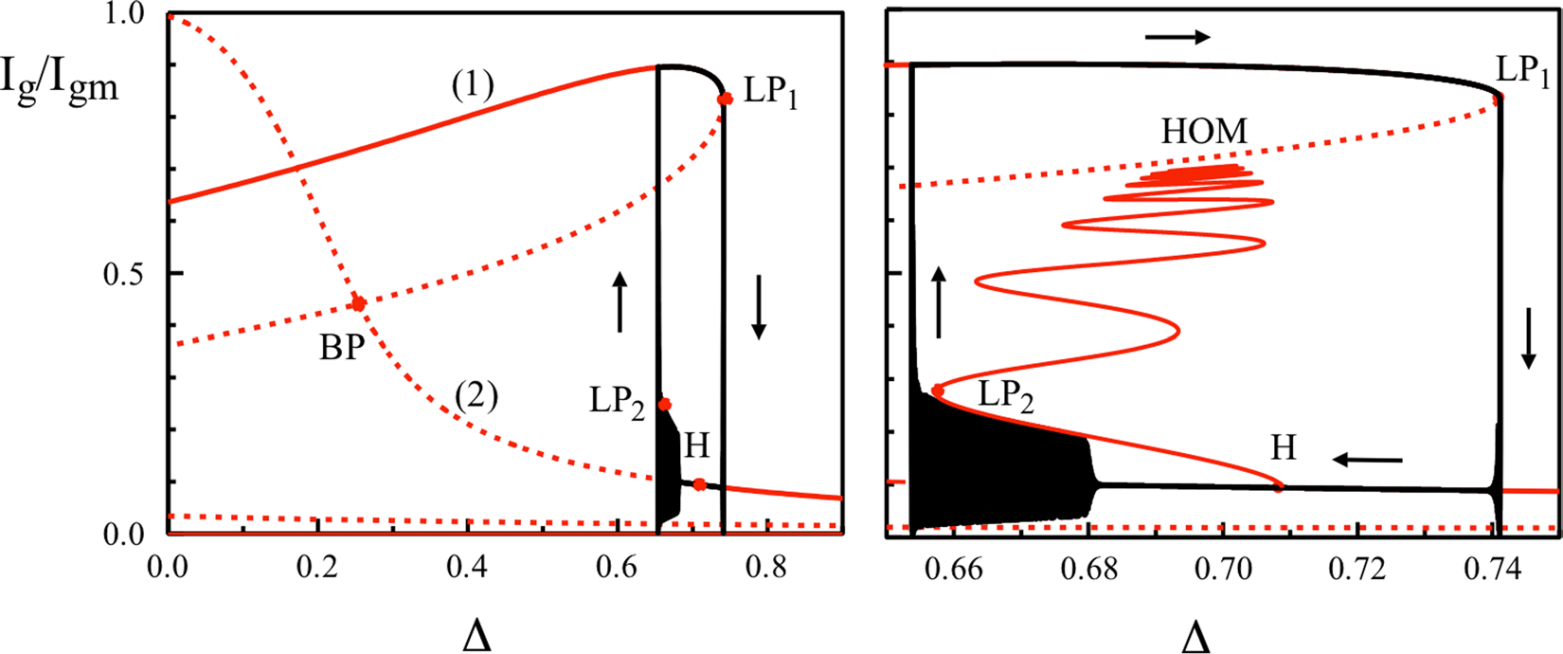
Left: The bursting cycle (black) in the phase plane (*I*_*g*_, Δ) is shown together with the bifurcation diagram *I*_*g*_ = *I*_*g*_(Δ) of the fast subsystem (1)–(5) (red). Line (1) corresponds to the branch of steady states where *I*_*g*_ ≠ 0 and *I*_*e*_ = 0. Line (2) represents the branch of steady states where *I*_*g*_ ≠ 0 and *I*_*e*_ ≠ 0. It emerges at the bifurcation point BP. Stable and unstable branches are shown by full and broken lines, respectively. The points LP_1_ and H mark a limit point of steady states and a Hopf bifurcation point, respectively. Right: The bursting cycle is shown together with the branches of steady and periodic solutions. The branch of periodic solutions represented by the maximum intensity is emerging from H and snakes with various stability changes until it reaches the unstable steady state branch at a homoclinic bifurcation point (HOM). The figure shows that the bursting oscillations follows a stable branch of periodic solutions until it reaches a limit-point of limit-cycles (LP_2_). *I*_*g*_ is normalized by its maximal value *I*_*gm*_ = 325.46. The fixed parameters are: ${B}_{e,h}={B}_{e,h}^{w}={C}_{h}={C}_{h}^{w}={10}^{2}$, ${C}_{e}^{w}=0$, *C*_*e*_ = 13.5, *ε* = 7, *α* = 3, *β* = 2.4, *g* = 0.55, *J* = 55, *η* = 0.04, Δ_0_ = 0.7, *γ*_*th*_ = 7 × 10^−6^, and *c* = 0.0055.

In Eq. (6), ${\gamma }_{th}\ll 1$ measures the slow thermal relaxation (in MHz range), Δ_0_ is the detuning in the absence of optothermal effects and *c* is the amplitude of the thermal index change. Figures 4 and 5 show the evolution of the GS and ES intensities obtained from simulating numerically Eqs (1)–(6). These are in qualitative agreement with the experimental traces. Figure 4 also shows the evolution of the detuning through the cycle. The slow evolution is clear as is the change in direction corresponding to the large intensity change. We wish to determine the physical mechanism leading to a complete bursting cycle and propose to analyze the solution of Eqs (1)–(6) in the phase plane (Δ, *I*_*g*_) taking account that *γ*_*th*_ is small.

To this end, we first consider the case *γ*_*th*_ = 0. The detuning Δ is then a constant in first approximation and motivates the bifurcation analysis of the fast subsystem (1)–(5) with Δ as the bifurcation parameter. The bifurcation diagram of the steady and periodic solutions is shown in Fig. 5 together with the long time solution of the full equations (1)–(6) (in black). LP1 denotes a limit point of steady states above which the SL unlocks from the ML. H marks a Hopf bifurcation point where the dual GS + ES lasing state (with a low GS intensity) changes stability as we decrease Δ from a large value. The branch of periodic solutions that emerges from the Hopf bifurcation point has been computed using a numerical continuation technique. The bifurcation is clearly supercritical and leads to stable periodic solutions. However, this branch folds back at a lower value of Δ and further snakes until it reaches the upper branch of steady states. As the amplitude increases the branches are sequentially stable and unstable with a continuous increase of the period. The period becomes infinite at the homoclinic point HOM. Of primary interest for the description of the bursting cycle is the first folding point of the Hopf bifurcation branch which we denote by LP2 in Fig. 5. This point corresponds to a limit point of limit-cycles below which bounded oscillations are no longer possible. We are now ready to follow a complete bursting cycle (shown in black in Fig. 5). Starting at the upper branch of steady states (in red), the SL follows the steady state branch as Δ slowly increases until it passes LP1. It then drops down to the lower branch of steady states. Because the intensities are now low, Δ decreases according to Eq. (6). The SL follows the low GS intensity branch, passes the Hopf bifurcation point H, and then jumps to sustained oscillations. The unusual long delay of the Hopf bifurcation transition in the absence of noise is known^26^. After the effective transition to the oscillations, the laser follows a slowly varying branch of limit-cycle oscillations as Δ further decreases until it reaches LP2. The laser then jumps up to the upper branch of GS steady state. The delay of the jump near LP2 is more significant than the one near LP1. In both the experimental and numerical time traces (Figs 2(d) and 5(d)) we observe two or three oscillations appearing before the jump to the upper state. Figure 5 shows that the minima of these oscillations approach the low intensity saddle which may explain the increase of their periods before the jump. As soon as the laser reaches the high GS intensity steady branch, Δ begins to slowly increase following Eq. (6), starting a new bursting cycle.

## Discussion

The phenomenon uncovered here represents a new bursting mechanism with many similarities to bursting mechanisms in neural dynamics^1, 3^. There is however an important difference. The biological bursting oscillations admit an active phase that does not exhibit a Hopf bifurcation point. Instead, the active phase immediately displays sustained oscillations once the system leaves the silent phase. The system then follows a branch of stable oscillations until it reaches a homoclinic orbit. Here, the active phase starts at a stable steady state, experiences a long delayed transition to a branch of stable oscillations, and finally stops at a limit point of periodic solutions. The most natural way to control the number of spikes during a burst is by changing the position of the Hopf bifurcation point H in the *I*_*g*_ versus Δ bifurcation diagram. We have verified both experimentally and numerically that this is indeed possible by changing the injection strength. The control of the number of spikes during a burst is relevant for optical communications since, as mentioned, bursts can contain more information than single pulses. Similarly, the total period of a complete cycle can be modified by controlling the position of the limit point LP_1_.

Previous works have shown that the unique two-color emission of QD lasers is interesting for a number of applications and they have been studied under different operating conditions^19, 23, 27–29^. Here, we demonstrate that an optically injected QD laser admits size-controlled bursts of two-color activity. It constitutes a new bursting mechanism with both similarities and differences to those already described in the literature. The easy control and fast timescales suggest that it may prove useful for encoding information analogously to neuronal communication.
